# Perspectives About Racism and Patient-Clinician Communication Among Black Adults With Serious Illness

**DOI:** 10.1001/jamanetworkopen.2023.21746

**Published:** 2023-07-05

**Authors:** Crystal E. Brown, Arisa R. Marshall, Cyndy R. Snyder, Kristine L. Cueva, Christina C. Pytel, Sandra Y. Jackson, Sherita H. Golden, Georgina D. Campelia, David J. Horne, Kemi M. Doll, J. Randall Curtis, Bessie A. Young

**Affiliations:** 1 Cambia Palliative Care Center of Excellence at UW Medicine, Seattle; 2 Division of Pulmonary, Critical Care, and Sleep Medicine, Department of Medicine, University of Washington, Seattle; 3 Department of Bioethics and Humanities, School of Medicine, University of Washington, Seattle; 4 Department of Family Medicine, Center for Health Workforce Studies, School of Medicine, University of Washington, Seattle; 5 Department of Medicine, University of Washington, Seattle; 6 Department of Anesthesiology and Pain Medicine, University of Washington, Seattle; 7 Center for Army Analysis, US Army, Fort Belvoir, Virginia; 8 Division of Endocrinology, Diabetes, and Metabolism, John Hopkins University, Baltimore, Maryland; 9 Division of Gynecologic Oncology, Department of Obstetrics and Gynecology, University of Washington, Seattle; 10 Division of Nephrology, Department of Medicine, University of Washington, Seattle; 11 Justice, Equity, Diversity, and Inclusion Center for Transformational Research, University of Washington, Seattle

## Abstract

**Importance:**

Black patients with serious illness experience higher-intensity care at the end of life. Little research has used critical, race-conscious approaches to examine factors associated with these outcomes.

**Objective:**

To investigate the lived experiences of Black patients with serious illness and how various factors may be associated with patient-clinician communication and medical decision-making.

**Design, Setting, and Participants:**

In this qualitative study, one-on-one, semistructured interviews were conducted with 25 Black patients with serious illness hospitalized at an urban academic medical center in Washington State between January 2021 and February 2023. Patients were asked to discuss experiences with racism, how those experiences affected the way they communicated with clinicians, and how racism impacted medical decision-making. Public Health Critical Race Praxis was used as framework and process.

**Main Outcomes and Measures:**

The experience and of racism and its association, as described by Black patients who had serious illness, with patient-clinician communication and medical decision-making within a racialized health care setting.

**Results:**

A total of 25 Black patients (mean [SD] age, 62.0 [10.3] years; 20 males [80.0%]) with serious illness were interviewed. Participants had substantial socioeconomic disadvantage, with low levels of wealth (10 patients with 0 assets [40.0%]), income (annual income <$25 000 among 19 of 24 patients with income data [79.2%]), educational attainment (mean [SD] 13.4 [2.7] years of schooling), and health literacy (mean [SD] score in the Rapid Estimate of Adult Literacy in Medicine–Short Form, 5.8 [2.0]). Participants reported high levels of medical mistrust and high frequency of discrimination and microaggressions experienced in health care settings. Participants reported epistemic injustice as the most common manifestation of racism: silencing of their own knowledge and lived experiences about their bodies and illness by health care workers. Participants reported that these experiences made them feel isolated and devalued, especially if they had intersecting, marginalized identities, such as being underinsured or unhoused. These experiences were associated with exacerbation of existing medical mistrust and poor patient-clinician communication. Participants described various mechanisms of self-advocacy and medical decision-making based on prior experiences with mistreatment from health care workers and medical trauma.

**Conclusions and Relevance:**

This study found that Black patients’ experiences with racism, specifically epistemic injustice, were associated with their perspectives on medical care and decision-making during serious illness and end of life. These findings suggest that race-conscious, intersectional approaches may be needed to improve patient-clinician communication and support Black patients with serious illness to alleviate the distress and trauma of racism as these patients near the end of life.

## Key Points

**Question:**

Are experiences with racism associated with patient-clinician communication and decision-making among Black patients with serious illness?

**Findings:**

In this qualitative study of 25 Black patients with serious illness, epistemic injustice was the most common manifestation of racism. These experiences were associated with worsened mistrust and negative patient-clinician communication outcomes, with medical decision-making fractured by mistreatment and trauma.

**Meaning:**

This study found that epistemic injustice and intersectionality were key features of Black patients’ experiences with racism.

## Teaser

This qualitative study investigates experiences with racism and their association with patient-clinician communication and decision-making among Black adults with serious illness.

## Introduction

Racial inequities during serious illness and end of life (EOL) are well documented.^1^ In the last 6 months of life, Black patients were more likely to be hospitalized and admitted to the intensive care unit, receive mechanical ventilation and cardiopulmonary resuscitation (CPR), and die in the hospital, studies have found.^2,3^ This was associated with disproportionately high health care costs, a high symptom burden, and poor quality of life at EOL among Black patients.^4^ Spirituality and religion, health literacy, and mistrust have been used to explain why this higher-intensity care exists.^2,4,5^ For example, religious beliefs are cited as a reason Black patients may not desire prognostic information despite evidence suggesting otherwise.^6,7^ Additionally, low engagement in advance care planning (ACP) and hospice enrollment is often attributed to low levels of health literacy and related knowledge deficits.^7,8^ Traditional approaches to develop interventions addressing health disparities often ask Black patients to conform to White normativity and discount lived experiences and perspectives that are often marginalized by health care institutions. However, traditional, deficit-based approaches focused on changing patient personal attributes burden patients and their caregivers to correct health inequities rather than improving the trustworthiness of health care workers (HCWs) and institutions.

High-quality, serious illness communication supports patients and caregivers by clarifying values and goals and is essential for effective shared decision-making.^9,10,11^ However, Black patients experience HCW bias, lack of physician diversity, and poor-quality patient-clinician communication.^12,13,14^ Research on Black patient experiences during serious illness and EOL often lacks critical, race-conscious approaches to account for lived experiences within racialized medical settings. This contributes to poor understanding of how these experiences may be associated with communication with HCWs and medical decision-making during serious illness.^1,15^ Majoritarian framing of health disparities is associated with pathologizing of Black patients and further widening of the equitable care gap.^16,17,18,19^ In contrast, critical, race-conscious approaches challenge disciplinary norms and actively work against pervasive practices and thinking that uphold white normativity by prioritizing narratives that counter mainstream medical viewpoints.^20,21^ A race-conscious approach centers the role of racism and racialized experiences of Black patients and the association of these experiences with Black patient beliefs and behaviors within clinical settings.^22^ This contrasts with typical, race-agnostic approaches that overlook the unique experiences shaped by historical and current structural and interpersonal racism and that often compare Black patient experiences with those of White patients without exploring the diversity of experiences and perceptions among Black patients themselves.

We conducted semistructured interviews with Black patients with serious illness to explore how racism may be associated with patient-clinician communication and decision-making. Public Health Critical Race Praxis (PHCRP), a pragmatic form of Critical Race Theory, provided framework and processes through the use of 10 principles within a 4-stage research process to acknowledge how Black patients who were marginalized and othered navigated medical settings^20,22,23,24^ (Table 1 and Table 2).

**Table 1.** Public Health Critical Race Praxis: Foci and Iterative Application to the Study^a^

Focus	Definition	Principles	Application
Contemporary patterns of racial relations	Describe racism and racialization as they operate in the current day	Primacy of racialization Race as a social construct Ordinariness Structural determinism	Authors acknowledged the backdrop of traumatizing and racist events, such as the police killings of unarmed Black people, increasing financial strain, and climate injustice and its disproportionate effects on Black patients
Knowledge production	Understand how racialization shapes research and beliefs about racial groups	Social construction of knowledge Critical approaches Voice	Authors examined how current research around Black patients with serious illness uses deficit-based frameworks and upholds white normativity, perpetuating stigma and bias, and advocate to move away from these racist approaches
Conceptualization and measurement	Define a study’s racism constructs in relation to social context	Race as a social construct Intersectionality	Authors elicited and acknowledged patients’ multiple marginalized identities and how these interlocking identities shaped their views about clinicians and their health care
Action	Using knowledge obtained to disrupt racial inequities	Critical approaches Disciplinary self-critique Intersectionality Voice	Authors moved beyond simply describing and disseminating results and are directly applying findings into an adaptation of a dual-facing program for clinicians and patients to address the distress of racism, facilitate racial healing, and promote accountability

^a^ Adapted from Ford and Airhihenbuwa.^23^ The original schematic has been modified to clarify how racism may be associated with interactions with health care workers and medical decision-making within racialized health care settings.

**Table 2.** Public Health Critical Race Praxis: Principles and Iterative Application to the Study^a^

Principle	Definition	Application
Race consciousness	Awareness of researchers’ racial positions	Authors clarified their biases and relationships with Black patients who were approached and enrolled in study participation, particularly as clinicians and researchers who were privileged and White adjacent
Primacy of racialization	Acknowledging that racial stratification is central to societal problems	Understanding the contribution of racial hierarchies within the racialized health care setting, including the shortage of Black clinicians
Race as a social construct	Significance that derives from social, political, and historical forces	Black race is a risk factor associated with specific racism exposures, such as poor patient-clinician communication, unconscious bias, and prejudice during serious illness
Ordinariness	Racism is embedded in the social fabric of society	Racism is ubiquitous; Black patients experience racism within racialized health care settings, and the experience of everyday racism is the underpinning of the interview guide
Structural determinism	Macro-level forces that drive and sustain inequities; the tendency of dominant group members to make decisions that preserve power hierarchies	Investigating power, social standing, and privilege that favor and protect clinicians and their decisions; eliciting perspectives of clinician hierarchies, especially within the context of a teaching hospital
Social construction of knowledge	Reevaluating knowledge within a discipline using antiracism methods of research	Dispelling beliefs that current quantitative research is objective and acknowledging that this knowledge is based on a position of white supremacy
Critical approaches	Dig beneath the surface of findings to develop a comprehensive understanding of one’s biases	Move beyond superficial existing knowledge and interpretation of mistrust in Black communities, especially positions that favor and uphold white supremacy
Intersectionality	Acknowledging the interlocking nature of concurrent social categories and the forms of social stratification that maintain them	Explicit investigation of how a patient’s other marginalized identities (sex, gender, sexual orientation, and being poor) multiply experience of racism and are associated with communication and decision-making
Disciplinary self-critique	Systematic examination by members of a discipline of its conventions and impacts on the broader society	Rejecting traditional, racist approaches to research and care of Black patients that uphold a deficit-based approach
Voice	Prioritizing the perspectives of marginalized persons and privileging the experiential knowledge of outsiders within	Authors purposefully chose to interview Black patients at an urban, county hospital to reach perspectives and lived experiences that are erased and viewed as “fringe” by majoritarian approaches to research

^a^ Adapted from Ford and Airhihenbuwa.^23^ The original schematic has been modified to clarify how racism influences interactions with HCWs and medical decision-making within racialized health care settings.

## Methods

### Study Design

This is a qualitative study of semistructured interviews using thematic analysis to elicit perspectives from Black patients with serious illness regarding the association of racism with communication and medical decision-making. This study was approved by the University of Washington Institutional Review Board. Participants provided informed consent. The study followed the Consolidated Criteria for Reporting Qualitative Research (COREQ) reporting guideline.

### Participants

We recruited Black patients with serious illness hospitalized at an urban academic county hospital in Washington State. Potential participants were aged 18 years or older, had no cognitive dysfunction, and had a serious illness defined as a diagnosis associated with a median life expectancy of 2 years or less (eTable 1 in Supplement 1).^25^ Patients were screened through the electronic health record (EHR) and approached on hospital day 2 or 3 for enrollment in an ongoing prospective study examining racism and communication. Wealth was measured by the sum of ownership of 5 assets (checking account, savings account, retirement funds, vehicle, and home ownership).^26^ The EHR was used to identify potential participants, but only patients who self-identified as Black were interviewed. After consenting, participants completed a survey containing the Discrimination in Medical Setting (DMS) scale,^27^ Group-Based Medical Mistrust scales,^28^ and Microaggressions in Health Care scales (Table 2).^29,30^ The Group-Based Medical Mistrust Scale contains 12 items measuring race-based medical mistrust on a 5-point Likert scale. It is scored by the sum of responses (range, 12-60), with higher scores indicating more mistrust.^28^ The DMS scale is a 9-item scale eliciting frequency of health care discrimination on a 5-point Likert scale. It is scored by the sum of responses (range, 6-30), with higher scores indicating more discriminatory events.^27^ The Microaggressions in Health Care Scale is a 6-item survey measuring patient-reported frequency of microaggressions from health care workers on a 3-point Likert scale. It is scored by the overall mean of responses, with higher scores indicating more microaggressions.^29,30^ Participants also completed the Rapid Estimate of Adult Literacy in Medicine–Short Form (REALM-SF), a measure of health literacy. Participants are given 7 medical words to read aloud. It is scored by the number of words correctly read.^31^ After completing the survey, participants were purposefully selected based on diagnosis, sex, and whether they endorsed experiencing discrimination on any DMS item. Participants were asked if they were interested in an interview to gather more in-depth perspectives on their experiences. Written informed consent was obtained for those who agreed to participate. If a patient was unable to write or was visually impaired, oral consent was obtained. Interviews occurred at bedside or via phone, depending on patient preference. A $20 gift card was provided for participation for both enrollment in the prospective cohort and the interview.

### Interview Guide

PHCRP was used to develop and pilot test the interview guide. This guide was refined to elicit perspectives around experiences with racism, communication, and decision-making (Table 3). PHCRP brings a critical, race-conscious approach to research by prioritizing perspectives of marginalized groups (centering the margins), rejecting deficit-based approaches (disciplinary self-critique), and considering other marginalized identities that multiply experiences of oppression (intersectionality). During interviews, patients were asked open-ended questions on experiences with racism, processing and coping with racism, the impact of racism on their health care experiences, and communication and decision-making as they neared EOL. Follow-up questions clarified how multiple axes of oppression shaped patient experiences.

**Table 3.** Interview Guide

Research question	Interview questions
What are patients’ experiences with racism?	Has there been a time when you felt you experienced racism or discrimination as a patient? Would you please share what happened?
	Have you seen others experience racism as a patient? If so, what happened? Do you know of anyone else who has been discriminated against by doctors or nurses?
	How does this affect your opinions about health care workers?
How do they process experiences with racism?	How did you make sense of that experience? What did you do? Who, if anyone, did you tell?
	Do you think there is anything else about you that makes your doctors treat or communicate with you differently? Please feel free to share experiences when doctors or nurses have said something to you that made you feel like they were treating you differently for another reason.
	You mentioned earlier [self-described or attributed identity]. I would like to hear more about how, if at all, you think that makes your doctors treat or communicate with you differently.
How are experiences associated with views of the health care system and beliefs about how clinicians care for them?	How, if at all, did this experience influence how you view the health care system? When you seek care? When and how you agree to procedures? When and how you advocate for your care?
	How, if at all, did this influence your view of health care providers?
How are experiences with racism associated with the way patients communicate with clinicians?	In what ways did this experience influence how you communicate with your provider?
	Is there anything you do different now or make sure to do when you talk with your providers? If so, what?
	Tell me how your past experiences interacting with doctors and nurses affect how you speak with the doctors caring for you.
	When you feel that your care is racist, biased, or discriminatory, how does that change the way you speak to your doctors and nurses?
	Is there anything that you wish you knew or had to better communicate with providers?
How are experiences with racism associated with decision-making toward EOL care?	What makes you more or less likely to follow a doctor’s or nurse’s recommendation when it comes to making decisions for your health care?
	If a doctor that is biased gives you a recommendation about what to do next for your health, how likely are you to follow their recommendations?
	How do your past experiences with racism and discrimination affect the way you make decisions for yourself when it comes to your health?
	How do your past experiences affect your decision to come to the hospital to seek medical care? Agree to a procedure that doctors recommend? Agree to doctors recommending against a procedure that they say won’t help you?
	How, if at all, do you think this experience influenced how you and your family make decisions about your care? About a breathing machine? CPR? A living will?

Abbreviations: CPR, cardiopulmonary resuscitation; EOL, end of life.

### Analysis

C.E.B., a Black and Korean pulmonary and critical care physician trained in mixed-methods research, and A.R.M., a multiracial, Asian and Pacific Islander research coordinator with experience in health care ethics and qualitative research, conducted all interviews. Lasting 30 to 70 minutes, interviews were conducted past the point of thematic saturation to ensure that breadth and depth of perspectives were captured. Thematic saturation was defined as the point at which no new information was discovered^32^ Interviews were audio recorded, deidentified, and transcribed. Interview transcripts were imported into DeDoose software version 9.0.86 (Sociocultural Research Consultants, LLC).^33^ Along with C.E.B. and A.R.M., C.R.S., a multiracial Black qualitative researcher with experience in health equity research and PHCRP, and C.C.P., a multiracial Hispanic research coordinator with experience in qualitative health research, reviewed transcripts and used open, inductive coding to produce a codebook. Preliminary themes and codes were reviewed, edited, and approved by the research team, including K.L.C., a Filipina American internal medicine resident. C.E.B., A.R.M., K.L.C., and C.C.P. independently coded an initial set of 3 transcripts before meeting to identify and reconcile discrepancies by refining existing codes or defining new codes for a final codebook. C.E.B., A.R.M., K.L.C., and C.C.P. coded remaining transcripts, with more than 60% (16 of 25 transcripts [64.0%]) undergoing a secondary review. DeDoose was used during initial open coding, codebook development and refinement of codes, and final stages of coding and secondary review.^33^

## Results

A total of 25 Black patients with serious illness (mean [SD] age, 62.0 [10.3] years; 20 males [80.0%]) were interviewed between January 2021 and February 2023 (Table 4; eTable 3 in Supplement 1).^26,27,28,29,30,31^ Participants overall had substantial socioeconomic disadvantage, with low levels of wealth (10 patients with 0 assets [40.0%]), income (annual income <$25 000 among 19 of 24 patients with income data [79.2%]), educational attainment (mean [SD] 13.4 [2.7] years of schooling), and health literacy (mean [SD] score in the REALM-SF, 5.8 [2.0]). Among patients with interviews, 6 patients died within 1 year of being interviewed. Among 43 patients approached for an interview, 12 individuals declined due to having no interest or feeling too ill. Other reasons included feeling unsure (1 patient), not wanting to further discuss racism (2 patients), or later changing their minds (2 patients); 4 patients agreed to be interviewed but could not be reached. Participants reported high levels of race-based medical mistrust and frequent experiences with discrimination and microaggressions from HCWs. We identified 3 broad qualitative themes: (1) experiences with racism, (2) epistemic injustice and mistrust, and (3) poor communication and mistreatment- and trauma-informed decision-making (Table 5). These themes are part of the insult accumulation of racism experiences (eTable 2 and eFigure 2 in Supplement 1), forming the lens through which participants experience the medical system and make medical decisions (eFigure 1 in Supplement 1).

**Table 4.** Patient Demographics and Indicators of Racism Experiences

Variable	Patients, No. (%) (N = 25)
Sex	
Males	20 (80.0)
Females	5 (20.0)
Age, mean (SD), y	62.0 (10.3)
Died within 1 y of interview	6 (24.0)
Diagnosis	
NYHA class III or IV heart failure	12 (48.0)
Charlson Comorbidity Index score ≥6	8 (32.0)
Metastatic cancer	2 (8.0)
COPD FEV_1_ <35% or O_2_ dependence	1 (4.0)
ESKD and diabetes or albumin <2.5 g/dL	1 (4.0)
≥Age 75 y^a^	1 (4.0)
Income, $/y (n = 24)	
<25 000	19 (79.2)
25 000-34 999	2 (8.3)
35 000-44 999	1 (4.7)
50 000-74 999	2 (8.3)
Years of schooling, mean (SD) (n = 24)	13.4 (2.7)
Religion	
Christian	17 (68.0)
None	6 (24.0)
Other	2 (8.0)
Insurance	
Medicaid	9 (36.0)
Private	8 (32.0)
Medicare	6 (24.0)
Other	1 (4.0)
Uninsured	1 (4.0)
Wealth (No. of assets)^b^	
0	10 (40.0)
1	4 (16.0)
2	8 (32.0)
3	1 (4.0)
4	2 (8.0)
REALM-SF score, mean (SD) (n = 19)^c^	5.8 (2.0)
Group-Based Medical Mistrust Scale score, mean (SD) (n =23)^d^	40.1 (8.3)
Discrimination in Medical Settings score, mean (SD) (n = 21)^e^	21.0 (7.5)
Microaggressions in Health Care Scale score, mean (SD) (n = 23)^f^	1.9 (0.6)

Abbreviations: COPD, chronic obstructive pulmonary disease; ESKD, end-stage kidney disease; FEV_1_, forced expiratory volume in 1 second; NYHA, New York Heart Association; O_2_, oxygen; REALM-SF, Rapid Estimate of Adult Literacy in Medicine–Short Form.

SI conversion factor: To convert albumin to grams per liter, multiply by 10.

^a^ Age 75 years or older diagnosed with 1 of the conditions associated with a median survival of 2 years, but of lesser severity^25^ (eTable 1 in Supplement 1).

^b^ Wealth is measured by the sum of ownership of 5 assets (checking account, savings account, retirement funds, vehicle, and home ownership).^26^

^c^ The REALM-SF is a measure of health literacy. Participants are given 7 medical words to read aloud. It is scored by the number of words correctly read.^31^

^d^ The Group-Based Medical Mistrust Scale contains 12 items measuring race-based medical mistrust on a 5-point Likert scale. It is scored by the sum of responses (range, 12-60), with higher scores indicating more mistrust.^28^

^e^ The Discrimination in Medical Settings scale is a 9-item scale eliciting frequency of health care discrimination on a 5-point Likert scale. It is scored by the sum of responses (range, 6-30), with higher scores indicating more discriminatory events.^27^

^f^ The Microaggressions in Health Care Scale is a 6-item survey measuring patient-reported frequency of microaggressions from health care workers on a 3-point Likert scale. It is scored by the overall mean of responses, with higher scores indicating more microaggressions.^29,30^

**Table 5.** Racism Experiences Among Black Patients With Serious Illness

Theme and topic	Patient quotes (participant No.)
Experiences with racism	
Systemic racism	“It’s too engulfed in racism. It’s too money driven and controlled by racist people with agendas” (8)
	“The treatment I’ve been given, it feels like I did something wrong” (6)
Personal experiences with racism	“One of the nurses, I had an IV right here that had a bump right here. I said to one of the nurses, ‘It infiltrated and came out.’ And she said, ‘What’d you say?’ And I said, ‘It infiltrated.’ And she said, ‘How’d you know about that?’” (4)
	“But yeah, this doctor. He was a doctor! He kept saying, ‘Yeah, what’s up boss! You okay, boss?’ Grab me or something, ‘You okay, boss?’ It’s just like, you know, we’re not street folks” (8)
Intersecting axes of oppression	“Yeah, my cousins, we will go and they, you know, they put you on a back burner a lot. You know what I’m saying? They go by not just your race, they go by your race and if you got the, I call it the ‘welfare insurance’” (12)
	“Because your doctors, the more they perceive you as educated, and the more they perceive you as being affluent—it’s a milking of the cow. I think that there are people that are making decisions for poor people that should not have been made” (5)
	“Instead, because of my insurance or something, I might not be accessing certain medical treatments, you know? And that sucks. I mean, I don’t know, everything is just designed for us to fail” (8)
Vicarious experiences	“Generally speaking, it’s really about the fact that the stereotype is drug use with people of color. And like extreme drug use that would cause heart issues. Like, and that’s not the case, and [my son] doesn’t do any mind-altering, fast kind of drugs, you know, and to think that that’s what they’re doing. It makes things worse. And it’s an assumed” (18)
	“Because I remember a time when we didn’t go to the emergency room. We didn’t go see a doctor. We did home remedies or whatever. Like my brother. He died of colon cancer. And that could’ve been, and if he went in time, that could’ve been stopped” (4)
Epistemic injustice and mistrust	
Dismissal of knowledge and beliefs	“I wish [doctors] would listen more and get over their god complex. I know I’m a patient, but they all got that god complex, like, ‘I went to school for this so I know.’ Well, this is my body, and I’m going through it” (21)
	“I was retaining fluid from my congestive heart failure. They basically said there’s nothing they can do, and they just said I could leave when obviously there was something wrong with me. You could see my body how I was swelled up. And they just turned me away. I went [to another hospital]. And they ended up admitting me, and they pumped out some 15 L of fluid out of my body” (23)
Double consciousness	“I’ve had a few things that I’ve seen that wasn’t right in the ER, and you know, when doctors conversate with you and act like you really less than. You get that feeling sometimes, and even sometimes at our clinics” (4)
	“I’ve been a bartender for 25 years, so I’m pretty good at reading people, body language, and things like that. So if doctors are looking down their nose at me, I can tell” (21)
	“I feel like I got a good judgment of people. I’m smart enough. I can see dry land through muddy water” (12)
Vulnerability	“I woke up a few hours later in MICU. I asked the doctor, ‘Where am I at?’ He said, ‘You’re in the MICU. Your blood pressure bottomed out. You about died.’ The tears rolled up. I [thought] about that doctor telling me to ‘go home,’ and it bothered me” (4)
	“[Speaking up] can make patients afraid of retaliation. Maybe a patient dies” (3)
Mistrust	“Well, the nurse comes and gives me the information from the doctor, so I can only trust the nurse so much as I trust the doctors, and I don’t trust doctors at all” (12)
	“I can’t see him making a rational decision in medicine because of his other views. I’m not valued, so your brain isn’t at 100% capacity trying to figure out what you can do for me to possibly cure me or heal me and send me on my way in better health than when I came. So I don’t see that in a person that puts energy into being racist. I can’t fathom them coming to conclusions that are good, medicinally speaking. I just don’t see it” (8)
Self-advocacy	“I always advocate for myself. It’s my body. It’s my life” (21)
	“I realize that I need to have my voice heard right when they try to treat me like that. That just makes me so angry” (1)
	“About a month ago, I walked out of here. Didn’t finish chemo[therapy]. Because I got 5 doctors in front of me, and ain’t none of them on the same page. You got one doctor want me to do this, one doctor want me to do that, another one do that” (2)
	“I don’t like to come across as if I know everything, but I’m always inquisitive. You know, pretend I’m stupid. I’d rather pretend I’m stupid than smart so I can gather as much information” (23)
	“I had a doctor that I was seeing and he was giving me a diagnosis, and I didn’t understand the diagnosis so I asked him for some studies on the diagnosis, basically peer-reviewed studies, and I read the studies” (5)
Communication and decision-making within mistreatment and trauma	
Limiting communication	“I choose my provider. And I’m very much more aware if they are going to be listening to me or talking at me and not trying to help me work out what I came there to do and get it worked out in my favor. I would choose to not talk to a lot of them” (17)
	“I don’t say anything sometimes because I don’t want to disrupt things or create a problem, even though there is a problem” (8)
Life-sustaining therapies	“There is always something to do because the medical community is very strong, very strong, and it has a lot of things that are coming in the hospitals to be used which are even better today than the human brain. And they are helping people to survive death. But those are not applied to people of color” (3)
	“I would like to live. I would like for them to do their best to keep me alive” (8)
Quality of life	“Those are things I definitely would be willing to discuss with doctors. In fact, the idea of do not resuscitate, the idea of, well, geez, if this happens, don’t do heroic measures, don’t feed me with a tube in this situation, yes! Because it’s, all of that is an impact on your dignity and your quality of life” (5)
	“You know, if I’m on that thing and I can’t come out of the slumber that I’m in, and it’s going to take a while or whatever and it never happens, pull the plug” (9)

Abbreviations: ER, emergency room; IV, intravenous; MICU, medical intensive care unit.

### Experiences With Racism

Participants described health care as an institution with substantial racism: “I always looked at [the medical field] as a racist, white supremacist–controlled institution,” a participant said (participant 8). Participants described personal experiences with discrimination, bias, and stereotyping. A participant recalled, “This [doctor] sent me home to die with congestive heart failure looking at her dead in the face”(participant 7). Another participant shared, “[This nurse’s] prejudice was her view of Black men, that I’m intimidating, loud, and aggressive, and she should be able to speak to me in any manner she wishes”(participant 8). Participants described discriminatory treatment based on multiple intersecting axes of oppression, particularly perceived ability to pay and insurance status. A participant said, “If you have lots of wealth, you get extremely good treatment. If you have less wealth, you get poorer treatment. If you aren’t of the right group, you get lousy treatment” (participant 5). Participants who were unhoused described difficult interactions with HCWs. A participant shared, “I’m homeless. So that limits me from really having a voice as it is. It doesn’t matter what I say” (participant 23). Another observed, “It’s hard for people to see me. Don’t let them say that you are homeless. That’s biased” (participant 15).

Participants reported vicarious experiences with racism that shaped their perspectives. One participant shared, “I have a cousin who has really bad anxiety. He goes to the hospital and they won’t give him anything because they think he’s just there for the drugs” (participant 11). Another participant recalled, “My daughter was having a baby and the anesthesiologist left her on the table to go give an epidural to the White lady in the next room” (participant 7). Participants described the vicarious mistrust associated with these experiences. A participant recalled, “They can turn off a pacemaker and restart it from miles away. [My brother] had concerns that somebody might decide to turn off his heart” (participant 5).

### Epistemic Injustice and Mistrust

Racism most commonly manifested as epistemic injustice, the refusal by HCWs to recognize knowledge and beliefs of participants because of their social identity.^34,35^ Participants described having knowledge about their bodies and experiences silenced and dismissed by HCWs:^34,36,37,38^ A participant said, “I’ve gone to the hospital and had people ignore what I’ve said because they thought I was uneducated. Some people assume that Black people are not capable of understanding what you’re saying or answering a question properly” (participant 7). Another participant noted, “If I tell a doctor, ‘No, it’s not that, it’s this,’ they think that you’re playing doctor. They don’t like you playing doctor” (participant 11). Another stated, “They take it as, ‘How dare you question me?’” (participant 23). These experiences were accompanied by a sense that HCWs were biased. “There is that element where you can tell, like you don’t expect me to know what you’re talking about. That’s kind of disappointing,” a participant said (participant 13).

Participants reported awareness of how HCWs viewed them, especially if they were poor, Black, or less educated. “We start to feel inferior, like I don’t want to ask [a question], because they’ll just laugh,” a participant said (participant 20). Care from HCWs with bias who devalued patients exacerbated feelings of vulnerability, especially when they saw how others, especially White patients, were treated. “There’s a lot of discrimination, and it doesn’t matter what I say. ‘Just do as I say.’ That’s the feeling that I get,” a participant said (participant 22). Another participant shared, “It’s a terrible feeling. I can’t stand up and walk away. And if I sit here and take this [expletive], my situation is going to get even worse” (participant 10). Participants worried about mistreatment or neglect in retaliation for voicing concerns about racism in their care. “I’m not going to say nothing. The last thing I’m going to do is start [expletive] with someone who’s going to decide whether I hurt or sleep well,” a participant said (participant 18).

When HCWs did not value or respect participants, the participants said they could not trust their care or medical decisions. One participant noted, “Trust is a very important factor in how you view your doctors and their supposed attempts to help you get well” (participant 8). Another participant stated, “If you don’t feel like you’re getting all the information, it’s hard for you to have faith and trust the doctors you’re working with. It makes you skeptical about the care you’re getting” (participant 7). Despite feeling vulnerable, some participants reported advocating for themselves. One participant shared, “I put it in a way to let them know that I’m not completely dumb and I do have a little bit of brain in my head” (participant 23). Other participants reported hypervigilance. “I’m really on guard. As soon as they say something like they talking to a 10-year-old, I’m coming with it. You’re not going to think you’re talking to a 10-year-old no more,” a participant said (participant 12) Many participants reported limiting their hospital stays or finding another clinician or hospital where they might feel safer. “I pretty much just go along with what they say until I hear something that doesn’t make sense to me. That’s why I try to keep my stays to a minimum, because the longer you stay, the more complicated things become,” a participant said (participant 23). Some participants reported relying on support persons or other sources to verify information that HCWs provided. A participant shared, “When it’s too much and it really feels like I’m being ignored, I call my best friend. Some of the doctor terms I don’t understand. I turn around and ask my best friend” (participant 1). Another stated, “Google a medication you hear the doctor say. If they say something is antibiotics and they’re not antibiotics, I’m going to keep quiet and get the [expletive] out of here before I die” (participant 24).

### Poor Communication and Decision-Making in the Context of Mistreatment and Trauma

Participants often felt that HCWs did not value their perspectives and could not be trusted, which was associated with poor communication. One participant stated, “The doctor could care less about having a relationship with you. He walks out that door, and he done forgot your name that fast” (participant 12). Some participants purposefully limited communication when sensing disregard from HCWs. “Maybe you don’t even tell them when you’re sick or you have a problem. You just kind of look after yourself. Your communication with that doctor isn’t very good,” a participant said (participant 11). Another participant stated, “I just get a feeling about them. They seem to be pushy or arrogant, so I just back off and let them do their thing” (participant 21). Some participants reported compensating for poor communication by trying to mitigate stereotype threat, the fear of conforming to a negative racial stereotype.^39^ Participants reported that these strategies were born from previous negative health care interactions. “I’ll talk to a doctor or social worker. I know how to navigate my way through the system, so to speak,” a participant said (participant 23). Other participants reported using code-switching, adjusting how they spoke to HCWs to fit the white-dominant medical culture.^40,41^ “I know how to rearrange my conversation, how to talk to them,” a participant said (participant 15), while another participant stated, “When in Rome, do as the Romans” (participant 12).

Given that communication with HCWs was compromised, many participants reported that decisions to take a medication or a doctor’s recommendation hinged or whether the clinician was trustworthy. While racism was widely endorsed, participants struggled to determine whether their doctor was racist. A participant shared, “I always ask them what’s in [the pills], why is it given to me? Sometimes, I wonder if I should even be taking them. But in medical school, they might’ve been taught that that’s what you do” (patient 8). Another participant shared, “I do tend to follow recommendations. I don’t believe when there’s unconscious bias that there’s a conscious intent to do harm” (participant 5).

Participants largely endorsed trusting physicians who recommended a procedure more than those who did not. “When it comes to a procedure they’re saying I don’t need and somebody else is saying I do, I’m going to believe the person saying I do,” a participant said (participant 12). Another patient stated, “God basically using these people to say, ‘If you don’t do this surgery, you’re going to die.’ The Bible says, ‘Thou shall not kill,’ so we shall not kill ourselves” (participant 2). Participants were open to discussing EOL care, but many expressed difficulty imagining EOL care that did not consist of receiving life-sustaining therapies. A participant shared, “They ask me, ‘If your heart stops, do you want me to revive you?’ Bring me back! I don’t want to go home on no mistake” (participant 9). Some participants reported wanting to maintain quality of life and dignity as they neared EOL but struggling with discerning a clinician’s recommendations to limit care apart from their role in a racist institution. A participant shared, “Am I prepared to trust what’s left of the quality of my life to this person? The quality of your life is not going to be fixed by trusting somebody who does something bad to you. I struggle with that” (participant 5).

## Discussion

In this qualitative study, we described 3 themes shared by Black patients regarding the association of racism with communication and decision-making during serious illness: racism experiences, epistemic injustice and mistrust, and poor communication and mistreatment- and trauma-informed decision-making. Findings did not differ by age, diagnosis, sex, income, or insurance status. Our study findings framed personal attitudes, beliefs, and behaviors not as deficits, but as responses to everyday bias, discrimination, and other forms of racism. Strategies and coping mechanisms reported by participants in our study were framed by HCWs and systems as behavioral shortcomings through coded, biased, and covertly racist language (eg, “noncompliant”).^42^ Conversely, participants in our study described these behaviors as strategies and acts of resistance to oppression. This study expands upon existing knowledge by identifying and describing epistemic injustice as a barrier to equitable communication and care for Black patients during serious illness and EOL.

Increased focus on inequitable access to high-quality palliative care has prioritized research that addresses the challenges of palliative care implementation for socially disadvantaged populations.^43,44^ Palliative care inequities among Black patients are largely associated with ineffective serious illness communication and ACP.^4^ Best practices around serious illness communication often focus on eliciting patient values, preferences, and goals of care.^45^ However, these frameworks assume that a patient has had fair access and equitable treatment (eFigure 2 in Supplement 1).^46,47,48,49^ High-quality serious illness communication has been associated with improved quality of EOL care, less crisis-oriented decision-making, and improved completion of ACP documents.^50^ For example, recommendations for patients with serious respiratory illness endorse engaging patients in values-based discussions about their preferences, wishes, and goals of care to better understand patient values and preferences without concern over controlling influences.^45^ However, our study findings suggest that Black patients who were multiply marginalized may have had values and preferences borne from surviving unjust conditions and unequal power relationships. Study participants had difficulty extrapolating current treatment preferences to unfamiliar future circumstances, a known difficulty for patients and surrogate decision-makers.^51,52^ Indeed, participants in our study struggled imagining care that did not consist of aggressive EOL therapies, with many voicing concerns that these therapies would be withheld because of the participant’s race. Even as some participants neared EOL, they continued to navigate concerns about subjective limitations of treatment options. W.E.B. Du Bois described “double consciousness,” the sense of always looking at oneself through the eyes of others.^53^ Indeed, participants described “multiple consciousnesses” and maintained hypervigilant states by continuously considering how HCWs viewed their behavior and presence and whether this affected the medical choices they were given.^54,55^ Hence, patients process information and partake in decision-making within the context of intertwining and intersecting axes of oppression that exacerbate poor health care experiences during serious illness and at EOL. Supporting patients during conversations around medical decision-making with trusted clinicians, especially those who are race concordant, may help patients make decisions based on personal values and preferences unencumbered by concerns of bias.^10,12^ Indeed, improving workforce diversity is crucial to addressing these disparities given that race concordance for Black patients has been associated with improved health and health care outcomes, including mortality.^10,56,57^

Suggested approaches to reduce palliative care inequities include improving clinician cultural awareness and workforce diversity, in addition to patient-focused interventions associated with improved health literacy, reduced mistrust, and increased uptake of ACP documentation.^10,56,57,58^ Medical mistrust has often been attributed to egregious historical injustices, such as the Tuskegee experiment and other forms of scientific racism.^59^ However, participants in our study did not cite Tuskegee as a reason they did not trust the medical system; they cited the everyday silencing and smothering of their voices.^34,35^ Indeed, research that continues to use deficit-based approaches asks Black patients to change, embrace white normativity, and move toward practices that disregard their lived experiences.^60,61,62^ Critical approaches to research, such as PHCRP, bring race consciousness and equity to the forefront while illuminating disciplinary conventions within research that reinforce and perpetuate stigma and inequities.^22^ For example, a critical approach to improving patient-clinician communication among Black patients who are multiply oppressed may use trauma-informed approaches focused less on goals of care and more on supporting compassionate and nonjudgmental communication to build trust in individual clinicians, empower patients, balance power dynamics, and improve transparency.^63^

### Limitations

There are limitations in our study. First, this study took place within a single urban academic medical center. Second, the sample size was small, and most participants had congestive heart failure. Interview participants also reportedly felt subjectively well compared with patients who completed surveys but declined interviews. In addition, there may have been self-selection for participants more willing to discuss racism and certain experiences or perspectives. Third, Black female patients were underrepresented, and the unique, intersecting experiences and oppression Black females face is underdescribed. However, eligible patients were overwhelmingly male. Additionally, while this was not a limitation, other racial groups experience inequities at EOL but have their own racialized experiences. We caution against extrapolating reported racialized experiences of Black patients in our study to other populations.

## Conclusions

In this qualitative study, Black patients with serious illness reported experiencing racism as being silenced and excluded by HCWs. These experiences were exacerbated by other marginalized, intersecting identities. These findings suggest that critical, race-conscious approaches that move away from placing patients in deficit-based frameworks that uphold white normativity may be needed to help manage the distress of racism and improve communication and decision-making for Black patients experiencing multiple axes of oppression during serious illness.

## Notes

Supplement 1. **eTable 1.** Diagnoses Associated With a Median Expected Survival of 2 Years or Less **eTable 2.** Additional Patient Quotes on Experiences of Racism in Clinical Settings **eTable 3.** Individual Participant Characteristics **eFigure 1.** Study Flowchart **eFigure 2.** Racism Experiences of Black Patients With Serious Illness and at End of Life

Supplement 2.
Data Sharing Statement
