# Supplementary material for: Perspectives About Racism and Patient-Clinician Communication Among Black Adults With Serious Illness
Source: JAMA Netw Open. 2023 Jul 5;6(7):e2321746. doi: 10.1001/jamanetworkopen.2023.21746 (PMC10323709; doi:10.1001/jamanetworkopen.2023.21746)
Supplement: Supplement 1. — eTable 1. Diagnoses Associated With a Median Expected Survival of 2 Years or Less eTable 2. Additional Patient Quotes on Experiences of Racism in Clinical Settings eTable 3. Individual Participant Characteristics eFigure 1. Study Flowchart eFigure 2. Racism Experiences of Black Patients With Serious Illness and at End of Life [file jamanetwopen-e2321746-s001.pdf]

Supplemental Online Content

Brown CE, Marshall AR, Snyder CR, et al. Perspectives about racism and patient-clinician communication among Black adults with serious illness. *JAMA Netw Open*. 2023;6(7):e2321746. doi:10.1001/jamanetworkopen.2023.21746

eTable 1. Diagnoses Associated With a Median Expected Survival of 2 Years or Less

eTable 2. Additional Patient Quotes on Experiences of Racism in Clinical Settings

eTable 3. Individual Participant Characteristics

eFigure 1. Study Flowchart

eFigure 2. Racism Experiences of Black Patients With Serious Illness and at End of Life

This supplemental material has been provided by the authors to give readers additional information about their work.

© 2023 Brown CE et al. *JAMA Network Open.*

eTable 1. Diagnoses Associated With a Median Expected Survival of 2 Years or Less[25]

- Chronic Obstructive Pulmonary Disease with Forced Expiratory Volume in 1 second (FEV1) <35% predicted or oxygen dependence
- Restrictive lung disease with Total Lung Capacity <50% predicted
- New York Heart Association class III or IV heart failure
- Left Ventricular Assist Device or Implantable Cardiac Defibrillator with age 65 years or older
- Child's class C cirrhosis or Model for End-Stage Liver Disease>17
- Dialysis dependent renal failure and either diabetes or serum albumin <2.5
- Pulmonary Arterial Hypertension with 6 Minute Walk Test <250 feet
- Cystic fibrosis FEV1<30% predicted
- Aged 75 years or older with diagnosis of at least one of the life-limiting chronic illnesses noted above, although possibly of lesser severity
- Hospitalization from any case within the pasts 18 months with diagnosis of at least 1 of the life-limiting, chronic illnesses noted above, although possibly of lesser severity
- Aged 90 or older
- Charlson Comorbidity Index score ≥6

© 2023 Brown CE et al. *JAMA Network Open.*

eTable 2. Additional Patient Quotes on Experiences of Racism in Clinical Settings	
**Theme**	**Quote**
Stereotyping	"Well, the way I talk right now, and the stuff that comes out of my mouth, they talk to me like you know, he talks ratchet and like he's from the hood so he's kind of stupid until they get to talking to me. Like, he may talk ghetto, but he is not no dummy."(P16)<br><br>"Well, that's all the same when people assume that you're not as smart. They look at you kind of, and if you're a Black preacher a lot of times there a stereotype that you can talk well but you're not book smart. And that's not necessarily true. But even in talking to some of the Black ministers I know, when we go to the churches, they, I'm sorry I've gotten heated thinking about it because, like visitations in the hospital. They ask us questions. In the Black community, the preacher used to be expected to know about law and medicine and statistics, finances, and everything to help people get through their lives. We were expected to know that and when you go into a hospital and interact with white people, basically, doctors don't expect you to know all of that and there's a tendency to be talked down to. It's just the thing where, you don't understand the facts, you don't understand the science, you don't understand what we're talking about. Stay in your lane." (P5)
Epistemic injustice	"You want to know about my experience? I refused to take Dilaudid, first of all. When they gave me Dilaudid, the doctor started giving me Dilaudid. So, I was still in a lot of pain and I was telling her that I'm in a lot of pain. They came up with the assumption that I'm a dope fiend and I'm trying to get more Dilaudid. And they said that, so she brought it down to 0.2 Dilaudid. And I told her that 0.2 Dilaudid is not helping me with my pain, do you have anything else that you can give me? And she said, 'Well, I gave you 0.4, but I did that just one time to see if it helped you.' And it did, but she didn't give that to me anymore because there was an assumption I was trying to get it because I'm dopey." (P17)<br><br>"It was an experience where I was in pain and I went to the emergency room and they told me that I was basically just imagining it. And I went back again the next day and told them I was really in pain and I was crying and they laughed at me. The nurse was standing outside the door, talking to another nurse and they were laughing at the fact that I was in the room crying over what they thought was nothing. And as it turned out, I had an abscess going down the back of my neck and my shoulders, and I ended up in a coma for two weeks. But they laughed." (P11)

© 2023 Brown CE et al. *JAMA Network Open.*

Intersectionality	"Being a woman? Definitely being a woman of a bigger body. They discriminate against us. Because they say, oh they just need to lose some weight, or whatever, whatever, whatever." (P7)<br><br>"Let's say homeless services. I have the right as a veteran to homeless services, you know. If you say to me 'No' for no reason, you're going to expect a certain reaction from me if I'm Black or white. A white person says, 'Why? What do I need to do', whereas a Black person goes 'Well, [expletive] y'all, y'all [expletive] are racist.' I've been in situations where somebody would provoke that emotion and kind of wait for a response. Like, 'Oh, you're not gonna scream at me? Okay yeah, oh geez. I gotta do my job, Jesus Christ, I'm gonna get fired.' And you know, as I'm walking out the door, saying 'Oh Mr. XXX I found it! You come back!' I see that change. You know. When you give them what they expect, then they say 'Okay, bye, I did my job.' But when you don't respond the way they expect you to respond, they get worried that maybe they might get in trouble. Because you seem very controlled and you know what to do next." (P18)
Microaggressions	"But I'm saying this lady was referring to me as 'partner' and I kindly said, 'Ma'am will you please not refer to me as partner. Please address me by my name or patient.' And she started going in on me talking about how she was so nice to me earlier. She's like, 'You know, why do you have to talk to me like that and tell me how to talk? Wasn't I was so nice to you?' She said, 'Didn't I treat you nice earlier? Wasn't I kind to you?' I said, 'You were, but that doesn't mean start calling me some other name or something I don't want to be addressed as.' If I knew that lady was going to jump to security and all that, I would've just let her call me partner. If I knew addressing that, asking her to call me by my name or 'patient,' if I knew that it was going to turn into a fiasco, I wouldn't have said anything." (P8)<br><br>"COVID is running rampant through the hospital and now we have to be more protective. And if I got five people in my room ain't no telling which one of them have COVID. And COVID right now is so dangerous because something you can't see is taking millions of lives. And we gotta be precautious. And they thought I was being funny when I told them all to get the [expletive] out of my room. I wasn't being funny, I was being precautious. Because I got five of y'all in here. Who got COVID? I got both of my shots and my booster shots. I got HIV, a congested heart, and I got cancer? I'm not trying to adad anything to the list. So, I'm thinking the doctors would at least take that into consideration." (P2)

© 2023 Brown CE et al. *JAMA Network Open.*

Discrimination	"When the new roommate came in and she was like, 'Well, it's just too warm in here,' they immediately shut the heat off not even considering the other patient that was in the room. So then it's freezing in that room. We bring it to [the nurse's] attention. And [the nurse] wants to go back and forth with me about, saying, well, I need to check with engineering and I need to this and I need to that. And I'm like, well hold on, you didn't check for it for [the other patient] when you turned it off, so why are you checking for it to turn it back on?" (P17)<br><br>"I told them no, we're not going to do [surgery]. First of all, I don't trust them anymore because I felt like they showed me such disregard. And basically, and in all honesty, if I was one of their white counterparts, they would've never did me like that, or they would've at least gotten back to me. You know?" (P7)
Structural racism	"Visiting hours, they were nine to six and I asked him how is that equitable? There are people out there that work from 9:00 to 5:00 and then they come up here and they have to find parking. By the time they get upstairs, it's already 6 o'clock. How is that equitable? You're making people have to choose between coming to see their loved one or going to work and paying their mortgage." (P17)<br><br>"It's all the same, I have to say. It's the same because the doctors I've seen here, they're the same. White people. And sometimes there was, we see, [doctors of color]. Where are they? Sometimes we get Asian doctors. Those are the ones who support white people. Over and over again, they support white people." (P3)
Unconscious bias	"Unconscious biases, yes. In fact, I would say that it's amazing because sometimes even the people of color will respond to a white person faster than they will to me and I don't know why that is. That really confuses me. And I notice that the longer as I was there, they would respond with, in fact, they would want to engage in conversation with them. So I felt some bias there."(P8)<br><br>"Doctors, they gotta earn any door knocked down. 'Cause it's all about them. If they had it their way, they'll be acting like they don't even want to touch you. They really don't. If they know what's wrong with you already, all the doctors wanna do is come in, get a stethoscope, check your heart, tell you to breathe, and leave." (P16)
Vicarious experiences	And then you know, like with COVID, we always heard people that were line jumping Black communities. It's no secret that Blacks are... The coded language with the "inner city" you know, and so that's why, I've learned somebody earmarks treatment for the wealthier,

© 2023 Brown CE et al. *JAMA Network Open.*

	more sophisticated whites using technology. We've heard about it. They'll go in there and line jump." (P20)<br><br>"[My brother] said, 'Bro look at it man. Look here, I'm gonna give you the upper hand in this thing'. I said, what you mean? [near whisper] 'All that medicine they giving you? Google that [crap]. Google it. That way, you'll know what they talking about before they even start talking about it.' He said, 'You always supposed to be two steps ahead of them, not two steps behind. By telling them you two steps behind, that's letting them know that you don't know what you need. And they can just pull you along, string you along. But, when you Google? You do your homework? You can go back in there and tell them what they already know and let them know you own your job like they own their job." (P2)

© 2023 Brown CE et al. *JAMA Network Open.*

eTable 3. Individual Participant Characteristics			
**Patient**	**Age**	**Sex**	**Diagnosis**
P1	60s	Female	Charlson Comorbidity Index score ≥ 6
P2	40s	Male	Charlson Comorbidity Index score ≥ 6
P3	70s	Male	Age 75 with congestive heart failure
P4	60s	Male	Charlson Comorbidity Index score ≥ 6
P5	60s	Male	NYHA III or IV congestive heart failure
P6	70s	Male	NYHA III or IV congestive heart failure
P7	60s	Female	NYHA III or IV congestive heart failure
P8	50s	Male	Charlson Comorbidity Index score ≥ 6
P9	60s	Male	COPD <FEV1 35% or O_2 dependence
P10	50s	Male	NYHA III or IV congestive heart failure
P11	60s	Female	ESRD and either and serum albumin <2.5
P12	60s	Male	Metastatic cancer or inoperable lung cancer
P13	50s	Male	NYHA III or IV congestive heart failure
P14	80s	Male	NYHA III or IV congestive heart failure
P15	50s	Male	NYHA III or IV congestive heart failure
P16	60s	Male	NYHA III or IV congestive heart failure
P17	60s	Female	Charlson Comorbidity Index score ≥ 6
P18	50s	Male	NYHA III or IV congestive heart failure
P19	70s	Male	Charlson Comorbidity Index score ≥ 6
P20	50s	Male	NYHA III or IV congestive heart failure
P21	60s	Female	Charlson Comorbidity Index score ≥ 6
P22	60s	Male	NYHA III or IV congestive heart failure
P23	40s	Male	NYHA III or IV congestive heart failure
P24	60s	Male	Metastatic cancer or inoperable lung cancer
P25	80s	Male	Charlson Comorbidity Index score ≥ 6
NYHA, New York Heart Association; COPD, chronic obstructive pulmonary disease; FEV1, forced expiratory volume in one second; O_2, oxygen; ESRD, end stage renal disease			

© 2023 Brown CE et al. *JAMA Network Open.*

FIGURE LEGEND

eFigure 1: Study flowchart for participant screening and enrollment. Eligible patients were screened and identified in the electronic health record and approached for enrollment into an ongoing prospective cohort. Potential interview participants were subsequently approached for participation in this qualitative study.

eFigure 2: Life course model of racism experiences of Black patients and impact on communication and decision-making. We use an accumulation model with risk clustering to display the insult accumulation of racism experiences as described by Black patients with serious illness in the study.

© 2023 Brown CE et al. *JAMA Network Open.*

FIGURES

eFigure 1. Study Flowchart

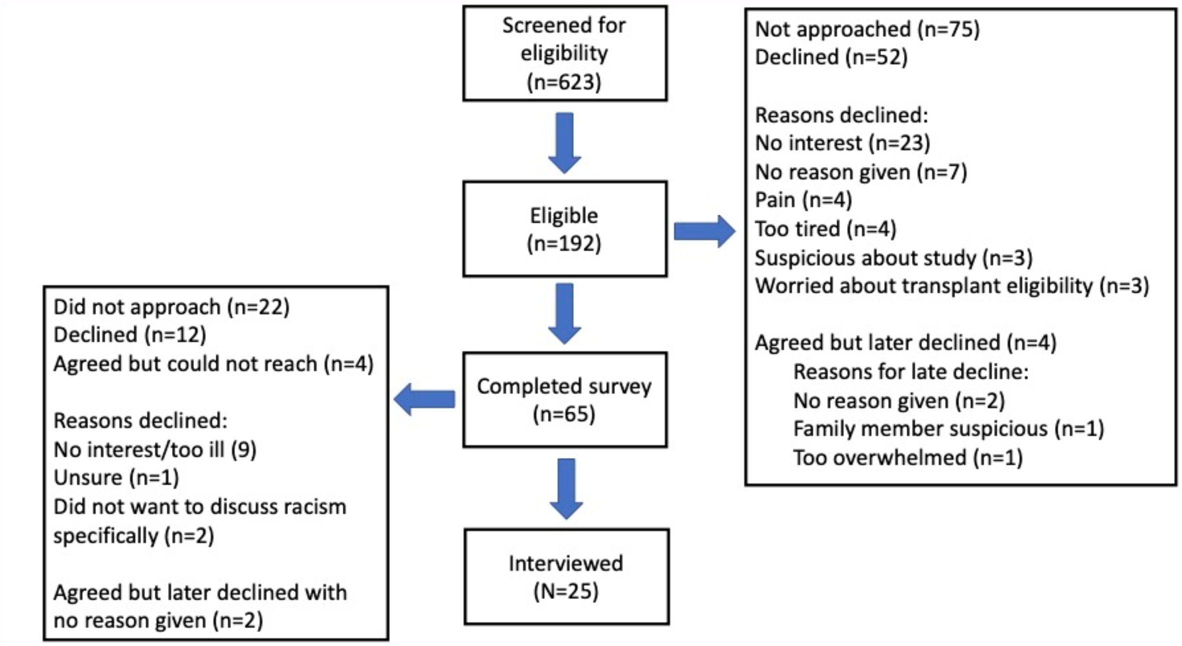

© 2023 Brown CE et al. *JAMA Network Open.*

eFigure 2. Racism Experiences of Black Patients With Serious Illness and at End of Life

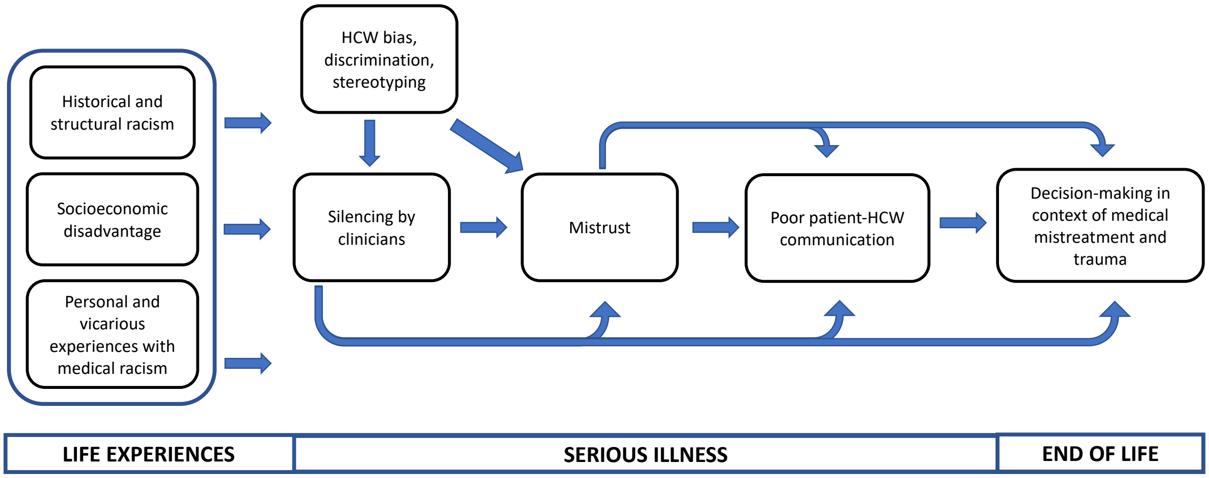

© 2023 Brown CE et al. *JAMA Network Open.*
