# Supplementary material for: Perspectives About Racism and Patient-Clinician Communication Among Black Adults With Serious Illness
Source: JAMA Netw Open. 2023 Jul 5;6(7):e2321746. doi: 10.1001/jamanetworkopen.2023.21746 (PMC10323709; doi:10.1001/jamanetworkopen.2023.21746)
Supplement: Supplement 2. — Data Sharing Statement [file jamanetwopen-e2321746-s002.pdf]

Data Sharing Statement

Brown. Perspectives About Racism and Patient-Clinician Communication Among Black Adults With Serious Illness. *JAMA Netw Open*. Published July 05, 2023.
doi:10.1001/jamanetworkopen.2023.21746

Data
Data available: No
